# Structural Basis of Cell Wall Cleavage by a Staphylococcal Autolysin

**DOI:** 10.1371/journal.ppat.1000807

**Published:** 2010-03-12

**Authors:** Sebastian Zoll, Bernhard Pätzold, Martin Schlag, Friedrich Götz, Hubert Kalbacher, Thilo Stehle

**Affiliations:** 1 Interfaculty Institute for Biochemistry, University of Tübingen, Tübingen, Germany; 2 Department of Microbial Genetics, Faculty of Biology, University of Tübingen, Tübingen, Germany; 3 Medical and Natural Sciences Research Center, Tübingen, Germany; 4 Department of Pediatrics, Vanderbilt University School of Medicine, Nashville, Tennessee, United States of America; University of California San Diego, United States of America

## Abstract

The major autolysins (Atl) of *Staphylococcus epidermidis* and *S. aureus* play an important role in cell separation, and their mutants are also attenuated in virulence. Therefore, autolysins represent a promising target for the development of new types of antibiotics. Here, we report the high-resolution structure of the catalytically active amidase domain AmiE (amidase *S. epidermidis*) from the major autolysin of *S. epidermidi*s. This is the first protein structure with an amidase-like fold from a bacterium with a gram-positive cell wall architecture. AmiE adopts a globular fold, with several α-helices surrounding a central β-sheet. Sequence comparison reveals a cluster of conserved amino acids that define a putative binding site with a buried zinc ion. Mutations of key residues in the putative active site result in loss of activity, enabling us to propose a catalytic mechanism. We also identified and synthesized muramyltripeptide, the minimal peptidoglycan fragment that can be used as a substrate by the enzyme. Molecular docking and digestion assays with muramyltripeptide derivatives allow us to identify key determinants of ligand binding. This results in a plausible model of interaction of this ligand not only for AmiE, but also for other PGN-hydrolases that share the same fold. As AmiE active-site mutations also show a severe growth defect, our findings provide an excellent platform for the design of specific inhibitors that target staphylococcal cell separation and can thereby prevent growth of this pathogen.

## Author Summary

Although Staphylococci are common habitants of the human skin and the respiratory tract, a number of highly pathogenic strains are a major cause of hospital-associated infections and can be life threatening especially in immunocompromised patients. Moreover, an increasing number of strains has acquired resistance against commonly used antibiotics, which makes treatment of infections a challenge. Therefore, antibacterial drugs that act on new targets are needed to counteract the further spread of multiresistent staphylococci. The autolysins, which are cell wall associated enzymes that are essential for cell proliferation, represent one promising such new target. We used x-ray crystallography to solve the structure of a catalytically active region of the autolysin of *Staphylococcus epidermidis*, AmiE, at atomic resolution. Our studies reveal a defined binding groove for a specific cell wall component on the protein surface. Using *in silico* calculations in combination with biochemical studies, we are able to identify key motifs that are required for the recognition of the cell wall by autolysins. Our data further indicate that, besides these core motifs, species-specific alterations of bacterial cell walls are responsible for the unambiguous identification of ligands. Knowledge of the interactions in an enzyme-substrate complex, as well as information about the mechanism of catalysis, are prerequisites for the successful development of antimicrobial drugs. Our results therefore provide a platform from which a new class of inhibitors can be launched.

## Introduction

Effective treatment of staphylococcal infections remains a worldwide challenge. In the United States alone, Staphylococci are responsible for about 19,000 deaths per year, a number that is higher than that associated with HIV [1]. The ubiquity of Staphylococci contributes to the constant emergence of new strains that are resistant to antibiotics. In particular, staphylococcal infections of immunocompromised individuals can lead to endocarditis, meningitis, pneumonia, septicemia and the toxic shock syndrome. Although many such infections are caused by *S. aureus*, the ability of the closely related *S. epidermidis* to form biofilms upon attachment to polystyrene surfaces poses serious problems during transplantation of medical prostheses [2]. The major autolysin AtlE (autolysin *S. epidermidis*) acts as key virulence factor in this process by mediating the initial attachment in catheter-associated infections [3]. It also binds to vitronectin, suggesting a role in colonizing host factor coated materials and host tissue [4].

**Figure 1:**
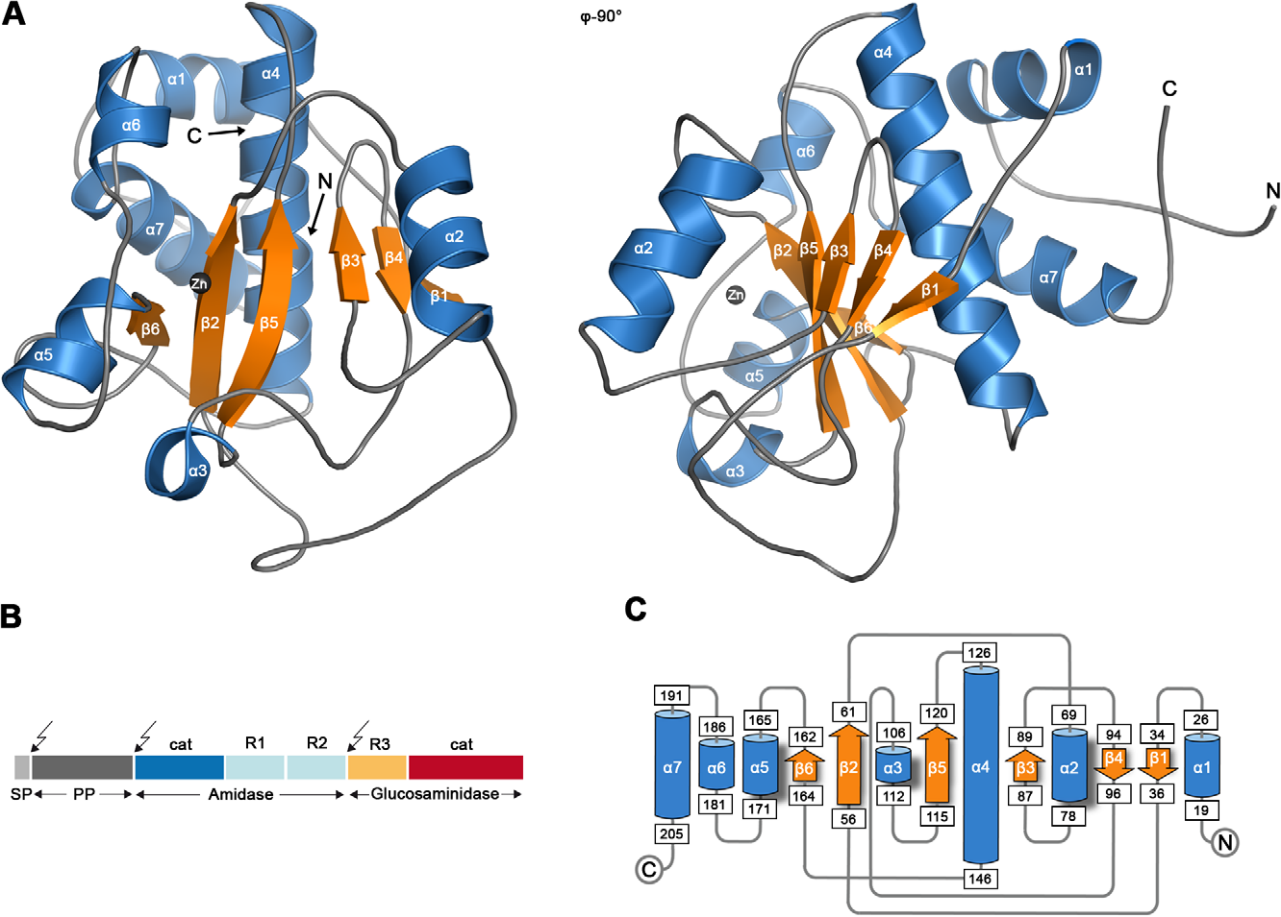
Crystal structure of the catalytic domain of AmiE. (A) Cartoon representation of the AmiE crystal structure. Two views differing by 90° are shown. Helices and strands are colored blue and orange, respectively. The zinc ion bound in the active center is shown as a grey sphere. (B) Domain arrangement of the bifunctional AtlE precursor protein. Arrows indicate the post-translational cleavage sites. **SP** signalpeptide, **PP** pro-peptide, **cat** catalytic domains, **R1 R2 R3** repeat domains. (C) Topology diagram of the AmiE structure, with helices and strands represented with cylinders and arrows, respectively. Numbers of amino acids are given in rectangles.

Together with the autolysin AtlA (autolysin *S. aureus*), AtlE belongs to a group of peptidoglycan (PGN)-hydrolases that play a pivotal role in the degradation of the bacterial cell wall [5],[6],[7]. During cell division, these autolysins are responsible for splitting the equatorial septum between two dividing daughter cells [8],[9]. Deletion mutants show a disordered division pattern with large cell clusters and were biofilm-negative [4],[7],[10]. The highly similar AtlA and AtlE proteins consist of a signal peptide, a pro-peptide, a catalytic domain with N-acetylmuramyl-L-alanine amidase activity, three repeats (R1-R3), and a C-terminal catalytic domain with N-acetylglucosaminidase activity (Figure 1B). After secretion, the precursor protein is processed extracellularly to yield the mature amidase (containing the catalytic domain and repeats R1R2) and glucosaminidase (containing repeat R3 and the catalytic domain) proteins. The amidase repeats R1R2 are responsible for attaching the enzyme to the cell wall but do not contribute to lytic activity [7],[11]. The catalytic domain, referred to as AmiE in *S. epidermidis* and AmiA in *S. aureus*, cleaves the amide bond between the lactyl moiety of N-acetylmuramic acid (MurNAc) and L-alanine in the PGN structure [7].

To provide a structural basis of autolysis function in *S. epidermidis*, we crystallized the catalytically active AmiE domain and solved its structure at 1.7 Å resolution. Using structure-based mutagenesis experiments and PGN digestion assays, we show that AmiE is a zinc-dependent metalloenzyme that requires a muramylpeptide with at least three consecutive amino acids as a substrate, while the side chain of the third amino acid can vary. Molecular docking provides evidence for extended contacts between the D-iso-glutamine (D-iGln) residue of the substrate and conserved residues in the putative ligand-binding groove of AmiE. These findings indicate an essential role of the glutamine isoform for substrate recognition. The structure therefore provides a framework for understanding substrate recognition, selectivity and catalytic mechanism of all staphylococcal amidases. It also reveals a striking and unexpected homology to the family of peptidoglycan recognition proteins (PGRPs), some of which also possess amidase activity. The AmiE fold closely resembles the PGRP-fold, including a conserved location of the active site and an asparagine in a conserved position likely to contact the second and third amino acid of the substrates peptide stem.

## Results

### Overall structure

The AmiE protein adopts a globular, mixed α/β fold, with a six-stranded, central β-sheet surrounded by seven α-helices (Figure 1A,C). The rear of the β-sheet packs against helices α4 and α7 and is shielded from solvent, while its front forms the bottom of a recessed area that is largely exposed and solvent-accessible. The recessed area, measuring about 28 Å by 10 Å, is walled off by helices α2, α3, α5, and several loops. Located at the center of the recessed area is a zinc ion that is held in place with contacts to side chains of residues H60 (β2), H165 (α5) and D179 (α5-α6 loop). The tetrahedral coordination sphere of zinc is completed by a water molecule, an arrangement often seen in the active sites of zinc-dependent metalloenzymes. Our structure revealed six additional bound zinc ions per monomer, which probably originated from the high concentration of zinc acetate present in the crystallization solution (see Materials and Methods). All six ions are located at or near surface loops. In order to verify that the zinc ion coordinated by residues H60, H165 and D179 is physiologic, we determined the structure of AmiE in a second crystal form grown without the addition of zinc-containing salts. In this crystal form, AmiE contains only the single zinc ion coordinated by H60, H165 and D179. The identity of this ion was confirmed by calculation of an anomalous difference fourier map (data not shown).

### Ligand binding site and enzymatic activity

**Figure 2:**
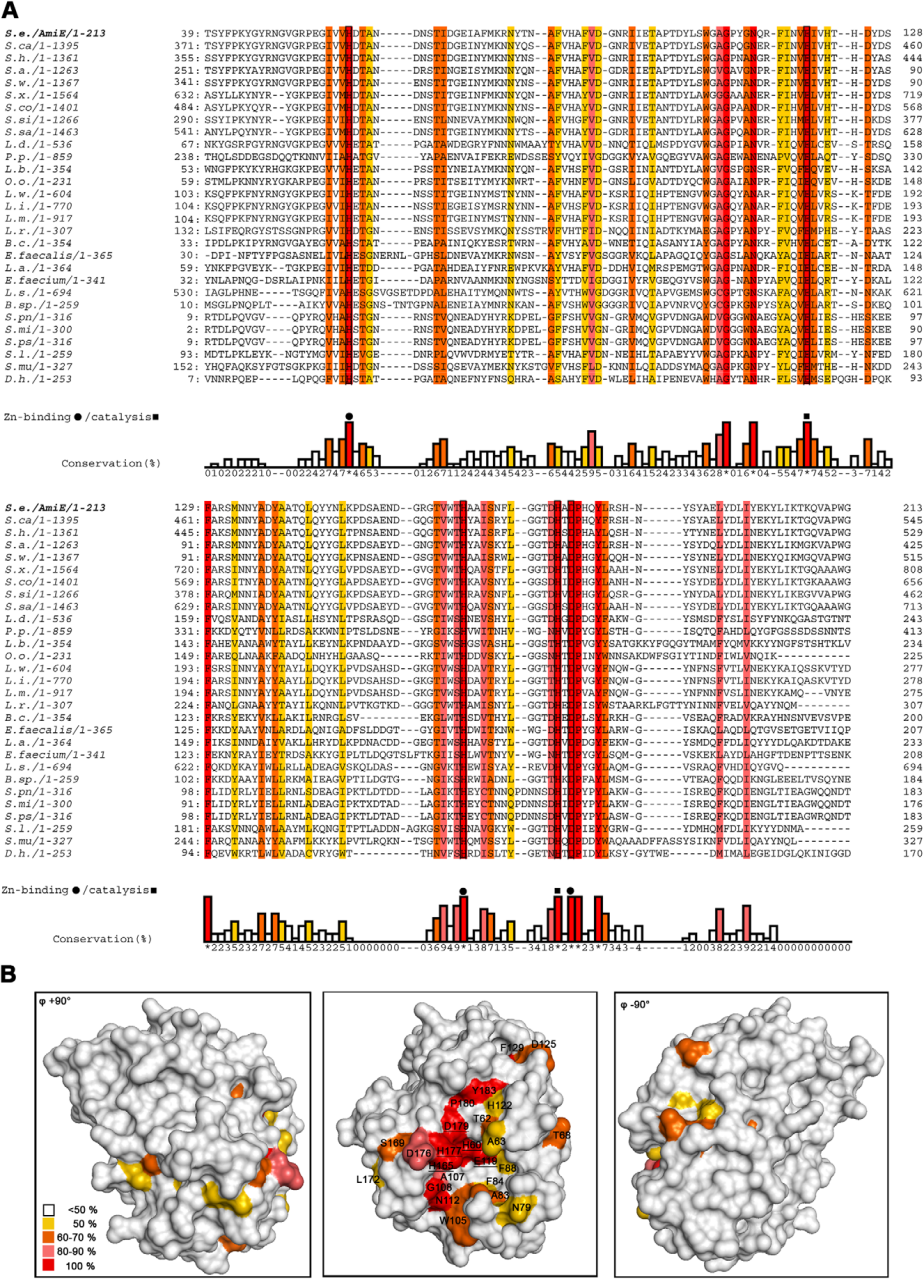
Conserved AmiE residues map to a single ligand-binding groove. (A) Sequence alignment of AmiE and 28 homologous proteins. Alignments were calculated with the programs ClustalW2 [34], MUSCLE [35] and MAFFT [36] and combined into a single output using COMBINE [37],[38]. Conserved amino acids are color-coded according to their degree of conservation, ranging from white (not conserved) to red (fully conserved). Residues participating in zinc-binding and catalysis are marked with rectangles and triangles, respectively. Abbreviations are as follows: **S.e** *Staphylococcus epidermidis*, **S.ca.** *Staphylococcus caprae*, **S.h.** *Staphylococcus haemolyticus* JCSC1435, **S.a.** *Staphylococcus aureus* RF122, **S.w.** *Staphylococcus warneri*, **S.x.** *Staphylococcus xylosus*, **S.co.** *Staphylococcus cohnii*, **S.si** *Staphylococcus simulans*, **S.sa.** *Staphylococcus saprophyticus*, **L.d.** *Lactobacillus delbrueckii* subsp. *bulgaricus* ATCC 11842, **P.p.** *Pediococcus pentosaceus* ATCC 25745, **L.b.** *Lactobacillus brevis* ATCC 367, **O.o.** *Oenococcus oeni* PSU-1, **L.w.** *Listeria welshimeri* serovar 6b str. SLCC5334, **L.i.** *Listeria innocua* Clip11262, **L.m.** *Listeria monocytogenes*, **L.r.** *Lactobacillus reuteri* F275, **B.c.** *Bacillus cereus* subsp. *cytotoxis* NVH 391–98, **E. faecalis** *Enterococcus faecalis* V583, **L.a.** *Lactobacillus acidophilus* NCFM, **E. faecium** *Enterococcus faecium* DO, **L.s.** *Lactobacillus sakei* subsp. *sakei* 23K, **B.sp.** *Bacillus sp.* B14905, **S.pn.** *Streptococcus pneumoniae*, **S.mi.** *Streptococcus mitis*, **S.ps.** *Streptococcus pseudopneumoniae*, **S.l.** *Staphylococcus lugdunensis*, **S.mu.** *Streptococcus mutans* UA159, **D.h.** *Desulfitobacterium hafniense* Y51. (B) Conservation pattern of amino acids on the surface of AmiE, shown in three different views. Amino acids are colored according to their degree of conservation using the color scheme of panel A. The majority of conserved residues, including residues with the highest degree of conservation, clusters in a distinct region around the catalytic zinc ion. Underlined letters mark amino acids of the coordination sphere (H60, H165 and D179) as well as H177 and E119, which have roles in catalysis.

**Figure 3:**
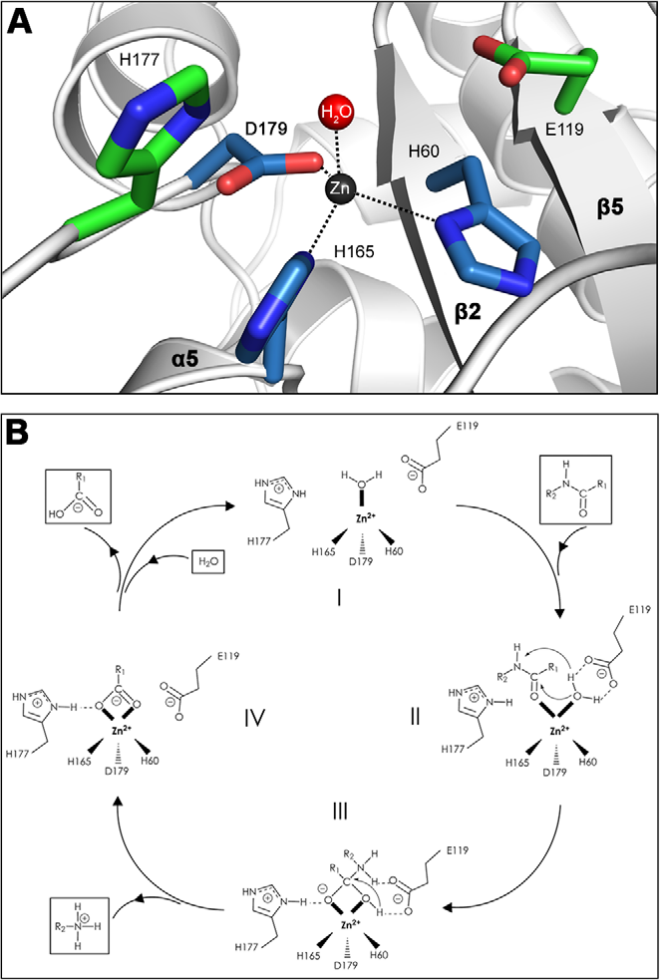
Close-up view of the AmiE active center and mechanism of catalysis. (A) Architecture of the active site. Side chains of H60, H165, D179 (blue) and a water molecule (red) coordinate a central zinc ion. Side chains of H177 and E119 (green) are 4.5 Å and 4.9 Å, respectively, apart from the zinc. E119 likely acts as a proton shuttle while the protonated side chain of H177 probably serves to stabilize a transition state. (B) Proposed mechanism of catalysis. The free enzyme is shown in (I). Upon docking of a PGN-fragment the Michaelis-Menten complex is formed (II). Acting as an electrophilic catalyst, the zinc ion accepts an electron pair from the carbonyl oxygen of the lactyl moiety, which becomes wedged between the water molecule and the side chain of H177. This results in a pentacoordinated zinc ion and a displacement of the water molecule towards the E119 side chain. The strong polarization between the positively charged zinc ion and the negative carboxylate of E119 leads to a nucleophilic attack of the water oxygen on the carbonyl carbon, which is in close vicinity. In this process, E119 serves as a proton shuttle by transferring the accepted proton to the nitrogen of the peptide bond. This results in the formation of a transition state (III), in which the former carbonyl carbon is now tetrahedral. The negative charge on the carbonyl oxygen in this state is stabilized by the protonated side chain of H177. In the next step (IV), E119 acts again as a proton shuttle by transferring the second proton. Thus, it promotes cleavage of the peptide bond and subsequent release of the peptide stem. In this state, MurNAc is still attached to the zinc ion via the lactyl carboxyl-group. Replacement against an incoming water molecule closes the catalytic cycle and reconstitutes the initial state (I).

Alignment of the AmiE sequence with 28 homologous bacterial proteins (Figure 2A) shows that only 10 out of 213 residues are strictly conserved in all sequences. Some of these are buried and likely contribute to the stability of the protein fold. However, the remaining residues cluster at the center of the recessed area, near the zinc ion (red residues in Figure 2B), and include the three zinc-coordinating residues H60, H165 and D179 (Figure 3A). Residues conserved to a lesser degree also cluster in this region, while the remaining AmiE surface is almost completely devoid of conserved amino acids. The conserved residues delineate a groove that runs along the recessed area, extending from the zinc ion to both the top and the bottom of the domain. The shape of the groove and its high degree of conservation indicate that it is the site of interaction with the PGN substrate. Moreover, the presence of zinc at the center of the groove suggests that AmiE functions as a zinc-dependent amidase. Residues E119 and H177, which are near the zinc ion but do not contact it, are likely to be involved in catalysis by facilitating a water-mediated nucleophilic attack at the peptide bond and stabilizing the transition state, as shown in Figure 3B.

**Figure 4:**
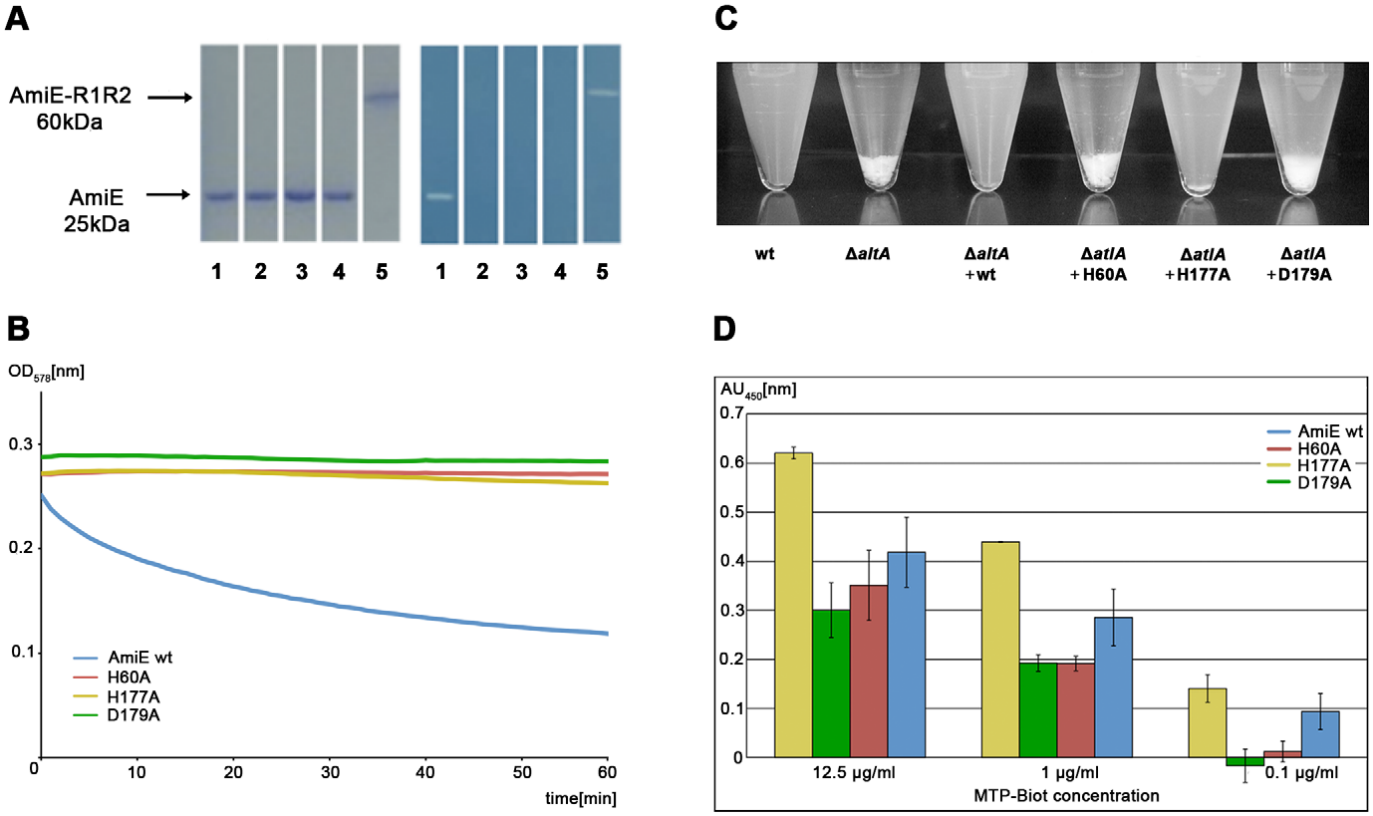
Activity and binding assays of amidase mutants. (A) Coomassie staining of the purified proteins after SDS-PAGE (left) and the corresponding methylene blue stained zymogram (right) with embedded heat-killed *S. aureus* cells. Lanes: **1.** wt AmiE, **2.** AmiE D179A, **3.** AmiE H177A, **4.** AmiE H60A, **5.** AmiE-R1R2 (control). (B) Digestion of *S. aureus* peptidoglycan in sodium phosphate buffer (100 mM, pH 7.0) at 37°C with 20 µg of the respective enzymes. Lysis of peptidoglycan was measured as the decrease in optical density at 578 nm. (C) Complementation of the *S. aureus* SA113Δ*atlA* mutant. SA113 wt and SA113Δ*atlA* mutant strains carrying different point mutations in the complementation plasmid pRC20 were grown for 10 h in B-broth. Sedimentation (aggregation) of cells was observed as a consequence of the inability of the cells to separate SA113 wt, SA113Δ*atlA*, SA113Δ*atlA* (pRC20_wt), SA113Δ*atlA* (pRC20_H60A), SA113Δ*atlA* (pRC20_H177A), and SA113Δ*atlA* (pRC20_D179A). (D) Binding affinities of biotin labeled MTP to inactive mutants. ELISA assay after 60 min of incubation with Streptavidin horseradish peroxidase at different MTP-Biot. concentrations. The best signal to noise ratio was obtained at a concentration of 12.5 µg/ml MTP-Biot.

In order to determine whether amino acids in the vicinity of the zinc ion are required for enzymatic activity, residues H60, H177 and D179 were separately mutated to alanine. All three mutant proteins were expressed and purified to homogeneity. None of them has lytic activity in zymogram gels with heat-inactivated *S. aureus* cells, whereas the wild type (wt) protein is able to lyse cell walls efficiently (Figure 4A). Similar results were obtained with lysis assays using purified *S. aureus* PGN (Figure 4B). Our results therefore provide evidence for a critical role of the zinc ion and its surrounding residues in catalysis. These findings are consistent with previous studies showing that treatment of the wt amidase with chelating agents such as EDTA or phenanthroline, which likely remove the zinc ion, results in loss of activity [12].

In order to assess the roles of amino acids H60, H177 and D179 *in vivo*, we expressed the wt gene coding for AmiE and repeats R1 and R2 (*amiE*-R_1,2_) as well as three mutated *amiE*-R_1,2_ genes carrying H60A, H177A and D179A substitutions in an *S. aureus* strain that lacks the autolysin (SA113Δ*atlA*, [7]) using the shuttle vector pRC20 [4]. The SA113Δ*atlA* strain has a severe growth defect, forming large cell clusters that quickly sediment in liquid medium. In contrast, the wt SA113 strain grows homogeneously and does not form such aggregates. As shown in Figure 4C, only the wt *amiE*-R_1,2_ gene is able to fully complement the Δ*atlA* mutant. The amidase mutants H60A and D179A are unable to complement the Δ*atlA* mutant, suggesting that these mutants are not functional *in vivo*. In comparison, the H177A mutant shows a milder phenotype *in vivo* and partially complements the Δ*atlA* mutant to wt phenotype. Residues H60 and D179 both coordinate zinc and play critical roles in catalysis (Figure 3). The H177 side chain is not part of the zinc coordination sphere, but most likely functions to stabilize the transition state during catalysis (Figure 3B). Our results thus indicate that only mutations directly in the active center are sufficient to abolish enzymatic activity, resulting in a phenotype that matches the deletion mutant. The H177A mutation possibly results in decelerated reaction kinetics, thus producing a less severe phenotype compared to those of the H60A and D179A mutants. The *in vivo* assay can be regarded as more sensitive since cells were grown for 10 h. The small time frame of the *in vitro* assay is not sufficient to discriminate between an enzyme with a reduced activity and no activity.

The lack of lytic activity in the three mutant proteins could be due to conformational changes that would adversely affect their interaction with ligand. To test this possibility, we generated the PGN-derived N-acetylmuramyl-L-alanyl-D-iso-glutamyl-L-lysine (MTP) ligand as previously described [13] and labeled it by attaching biotin to the ε-amino group of L-lysine via a short linker (MTP-Biot) (see Methods). Affinity measurements using an ELISA assay clearly show that all three catalytically inactive mutants are still able to bind MTP-Biot (Figure 4D). Compared with the wt protein, the H177A mutant even shows significantly increased binding. The most likely explanation is that the H177A mutation leads to a smaller structural change in or near the active site that facilitates binding. As with many enzymes, the AmiE active site is probably designed to preferentially bind and stabilize the transition state. Since H177 is likely to participate in this stabilization (Figure 3B), its replacement with alanine may reduce conformational stress on the substrate, allowing for better binding of the native conformation. It is also possible that the wt protein partially cleaves MTP-Biot, which would result in a lower signal. However, as variation of the incubation time does not affect the value for the wt protein (data not shown), we consider this latter possibility unlikely.

### Ligand specificity

**Figure 5:**
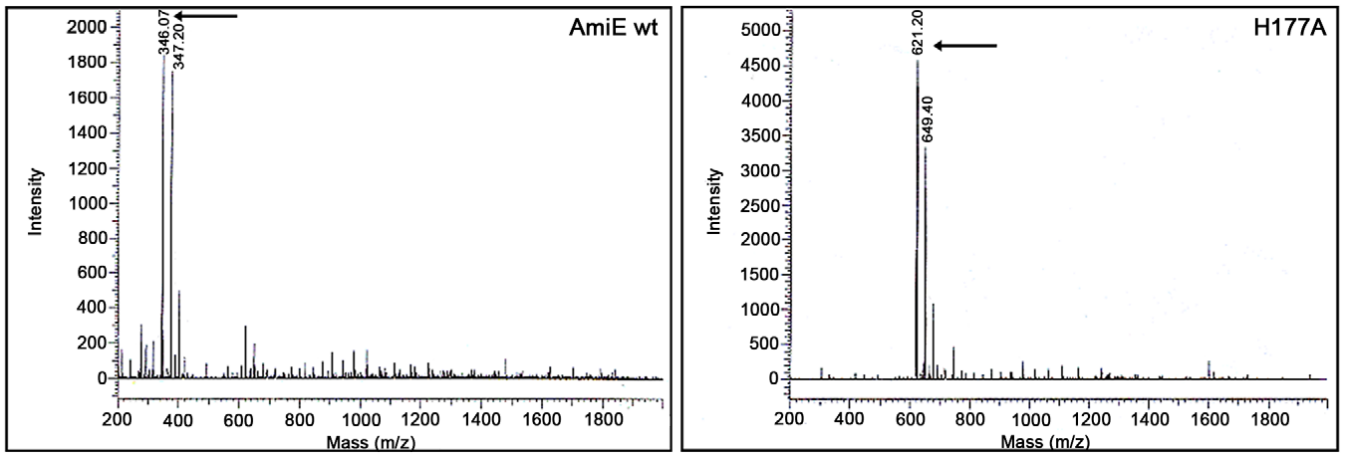
MTP digestion assay. ESI-MS spectra of MTP digest with AmiE wt and H177A. The wildtype enzyme and an inactive mutant (H177A) were incubated for 72h with MTP. ESI-MS spectrum of AmiE wt + MTP (left). Arrows indicate the masses of the digestion products. The main peaks are at m/z 346 and 374, which correspond to the cleaved peptide stem. ESI-MS spectrum of MTP + H177A (right). The m/z peaks at 621 and 649 correspond to MTP as synthesized.

To determine the minimal PGN sequence that can still be cleaved by AmiE, we performed cleavage assays with three PGN-derived compounds: MTP, N-acetylmuramyl-L-alanyl-D-iso-glutamine (MDP), and N-acetyl-D-glucosaminyl-(1,4)-N-acetylmuramyl-L-alanyl-D-iso-glutamine (GMDP). MDP and GDMP were purchased from Sigma and Calbiochem, respectively, whereas MTP was synthesized as described [13]. All ligands were incubated with the wt protein for 72 h at 37°C, followed by analysis of products with HPLC and ESI mass spectrometry. These experiments demonstrate that neither MDP nor GMDP are cleaved by the wt enzyme (data not shown), whereas MTP is cleaved into two fragments. The H177A mutant, which still binds to MTP, does not cleave MTP and therefore serves as a negative control in this experiment. The m/z profile of the H177A mutant is dominated by peaks at m/z 621 and 649 (Figure 5, right). The latter peak represents the MTP educt plus an unknown additional mass, which probably originates from the synthesis. Both peaks are replaced by peaks at m/z 346 and 374 in the profile for the wt protein (Figure 5, left). The first peak corresponds to the expected mass of the cleaved peptide stem while the second one is the digestion product of the compound with the higher mass. We conclude that cleavage by AmiE requires the presence of a third amino acid in the peptide stem. As the lysine is quite distant from the active site, it is most likely not required for the catalytic reaction itself but likely serves to anchor the ligand in the binding site. Similar cleavage results have previously been obtained for the catalytically active PGRP-L protein, which also requires MTP for cleavage [14].

### Structure-based molecular docking of MTP

**Figure 6:**
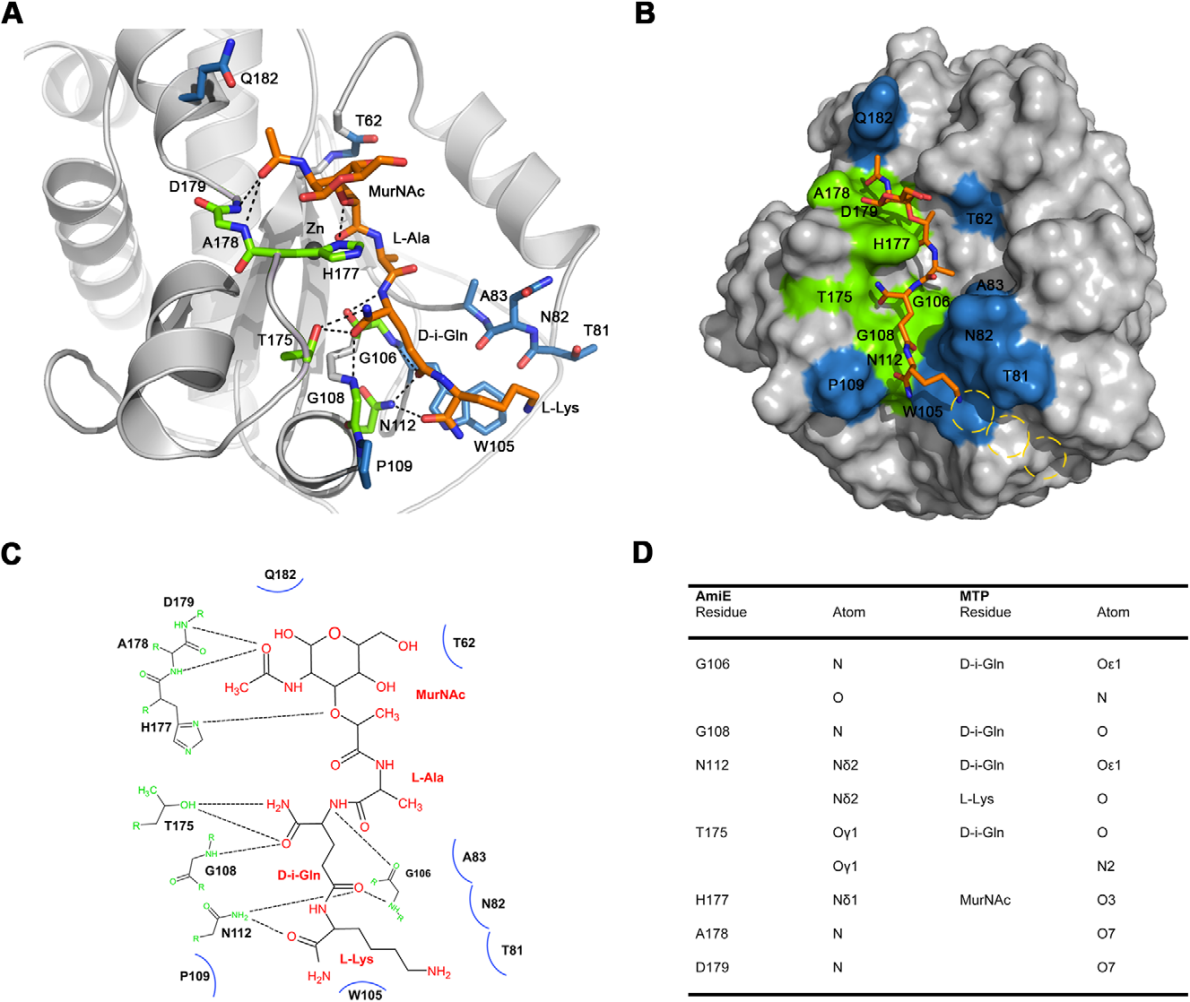
Results of docking of AmiE with the MTP ligand. (A) Putative interactions between AmiE (grey) and MTP (orange) in the docking model. AmiE residues forming hydrogen bond are colored green, residues making three or more van der Waals interactions are colored blue. Hydrogen bonds are indicated with dashed lines. (B) Surface representation of AmiE with MTP docked into the binding groove. Residues forming hydrogen bonds and van der Waals interactions were calculated using the PISA server (http://www.ebi.ac.uk/msd-srv/prot_int/pistart.html) [39]. The putative locations of glycine residues, which would be attached to the lysine side chain in the natural ligand, are indicated with yellow circles. (C) Schematic representation of interactions between AmiE and MTP. Van der Waals interactions are represented with arcs. The same color scheme for contacting residues was used in panels A, B and C. (D) Summary of the observed hydrogen bonds in the model.

Efforts to obtain a crystal structure of AmiE with a bound PGN fragment, either through co-crystallization or soaking, were unsuccessful. We therefore undertook molecular docking studies to establish a plausible mode of interaction for the MTP ligand with AmiE (see Methods). The available biochemical and structural data served as constraints for this approach. The best docking solution, which combines the highest docking score with a sensible orientation in the binding groove, is shown in Figure 6. The solution places the carbohydrate moiety in a relatively open, surface-exposed region at one end of the groove, and the tripeptide stem into the groove. Residues in the active site near the zinc ion, which are likely to play a role in catalysis, are positioned near the bond that is cleaved by AmiE. A nucleophilic attack of this bond by an activated water molecule, as shown schematically in Figure 3B, would therefore easily be possible.

Main chain and side chain atoms of residues in the second and third position of the peptide stem are able to make favorable contacts with residues in the AmiE groove. In particular, the model indicates a significant role for the D-iGln residue in substrate recognition as this residue forms 6 of the total 10 hydrogen bonds with the protein (Figure 6). Main chain atoms at both ends of the residue as well as side chain atoms are engaged in contacts. As equivalent contacts would not be possible with a standard glutamic acid residue, our model presents evidence for the importance of the glutamine isoform for substrate recognition. Asparagine 112 is strictly conserved among proteins that likely share the amidase-like fold (Figure 2) and occupies a position in the short α6 helix that allows the formation of hydrogen bonds between the side chain nitrogen and the two main-chain carbonyl oxygen atoms of D-iGln and L-Lys (see below).

We also note that, in our model, the L-lysine side chain fits into a surface pocket. The hydrophobic portion of its side chain forms extensive van der Waals interactions with the aromatic ring of W105. A prolonged lysine side chain with additional glycines of the interpeptide bridge would most likely run along the mainly hydrophobic chute that starts near W105 (Figure 6B, yellow circles).

### The influence of substrate modifications on catalysis

In agreement with the docking model, digestion assays with MTP suggest an important role of the third amino acid in the peptide stem. In order to further investigate the role of the lysine side chain for substrate recognition we performed additional digestion assays with MTP derivatives. For each of those, the sugar moiety (MurNAc) was replaced with a cleavable fluorescent (7-Methoxycoumarin-4-yl)-acetyl (Mca) reporter group since this group is known to have little effect on substrate recognition [12]. Substrates with one, two or three glycine residues attached to the lysine side chain were prepared (Mca-Ala-D-iGln-Lys(Gly)-D-Ala-Arg-OH, Mca-Ala-D-iGln-Lys-(Gly)_2_-D-Ala-Arg-OH, Mca-Ala-D-iGln-Lys(Gly)_3_-D-Ala-Arg-OH), as well as a compound in which the lysine is replaced with an alanine (Mca-Ala-D-iGln-Ala-D-Ala-Arg-OH). The exact number of glycines tethered to the lysine side chain of the natural PGN substrate recognized by AmiE is still unknown. Nevertheless, the chosen compounds match the *in vivo* conditions better than a terminal lysine side chain with a charged amino group. The Lys-substrate (without glycines) served as a reference.

**Figure 7:**
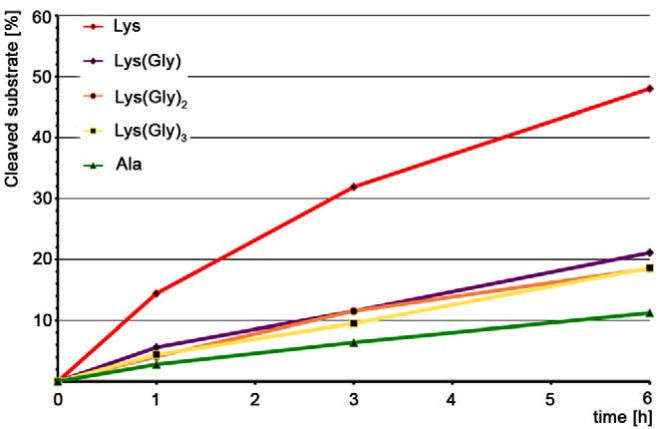
Digestion assay with modified AmiE substrates. Five substrates, differing in the modification of the third amino acid, were digested by AmiE over 6 h at 37°C. The amount of cleaved substrate was measured at the indicated time points.

Our results clearly show that all five substrates can be cleaved by AmiE (Figure 7). However, reaction kinetics are rather slow, which might be due to the fact that the substrates lack the N-acetyl-D-glucosaminyl and N-acetylmuramyl sugar moieties. Approximately 10 percent of the Ala-substrate is cleaved after 6 h. All of the Gly-substrates were processed similarly, with about 20 percent cleaved after 6 h. The Lys-substrate shows the fastest rate of cleavage, with almost half of the educt being digested within the same time. Comparison of the cleavage efficiencies between the Lys(Gly)_x_-substrates and the Ala-substrate reveals that the lysine side chain does not appear to have a significant impact for substrate recognition as the mutation of Lys to Ala only slows cleavage but does not prevent it. Furthermore, the negligible differences among the Lys(Gly)_x_-substrates show that an increasing number of glycines does not result in enhanced binding to AmiE. As our docking model shows, the charged ε-amino group of the Lys-substrate could easily form a cation-pi bond with W105 at the end of the PGN-groove (Figure 6A), leading to enhanced binding and perhaps accounting for the increased cleavage rate observed for this compound. Such an interaction would however not occur *in vivo*.

### Structural relationships

Although the amino acid sequence of AmiE does not exhibit significant homology to other structurally known proteins, we were surprised to find that its fold is strikingly similar to that observed in other zinc-dependent amidases. A DALI analysis [15] shows that the closest structural homolog of AmiE is the N-terminal domain of PlyL, a prophage endolysin encoded by the *Bacillus anthracis* genome (Z-score = 18.4, rmsd = 2.1) [16]. Like AmiE, PlyL is an N-acetylmuramyl-L-alanine amidase, cleaving PGN at the same site. Interestingly, the domain organization of PlyL, which contains an N-terminal catalytic domain and a C-terminal cell-wall binding domain, mirrors that of AtlE, which also has an N-terminal amidase followed by repeats that are reported to interact with components of the staphylococcal cell wall [7].

**Figure 8:**
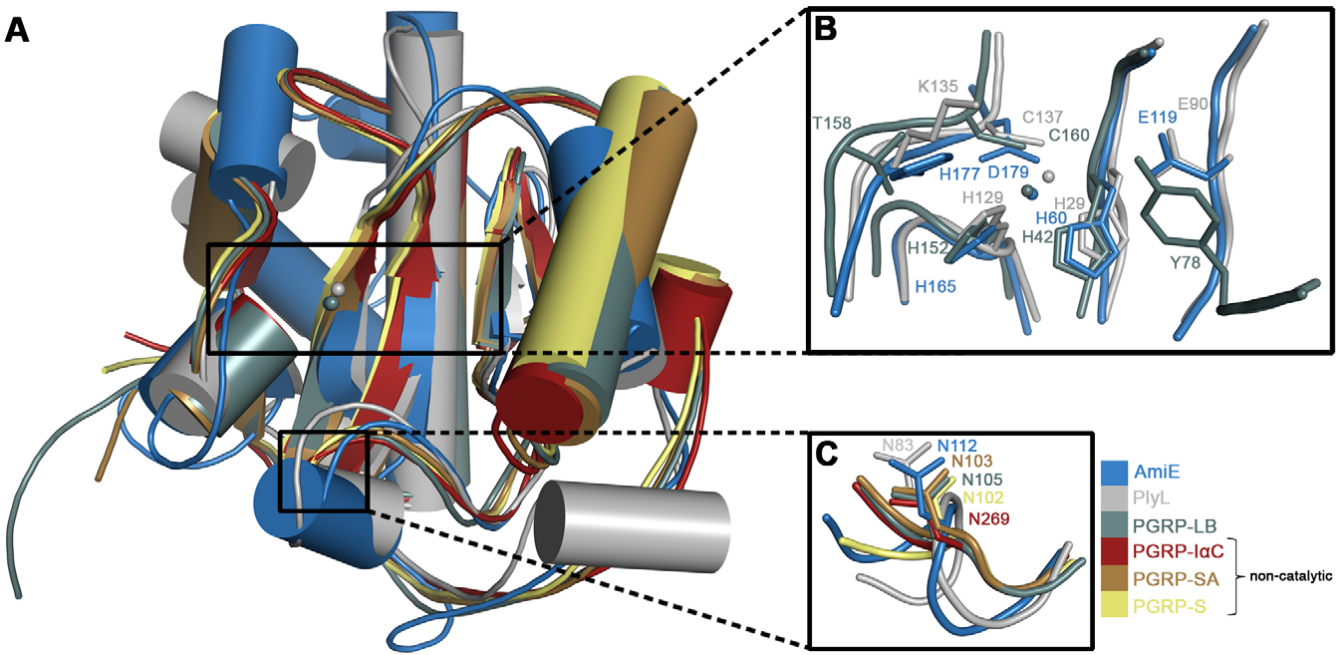
Structural comparison of AmiE with homologous proteins. (A) Representation of the overall fold. Helices are shown as cylinders, strands as arrows. Zinc ions in the active centers are shown as spheres. R.m.s. deviations and Z-scores were calculated by the DALI server [15]. PlyL (*B. anthracis* prophage) has the strongest structural homology to AmiE (Z-score = 18.4, rmsd = 2.1). This is followed by *D. melanogaster* PGRP-LB (Z-score = 12.4, rmsd = 2.3), *H. sapiens* PGRP-S (Z-score = 12.4, rmsd = 2.4), *H. sapiens* PGRP-IαC (Z-score = 12.3, rmsd = 2.3) and *D. melanogaster* PGRP-SA (Z-score = 12.1, rmsd = 2.4). Alignments were calculated with the program lsqkab from the CCP4 suite [23]. (B) Close-up view on the superimposed active sites. Zinc-coordinating residues and residues participating in catalysis are labeled and color-coded. (C) Superimposition of structurally conserved asparagine residues making contacts with the second and third amino acid in the peptide stem of MTP.

The AmiE structure is also highly homologous to the PGRP family of proteins (Figure 8). PGRPs are pattern recognition molecules that are conserved both in vertebrates and invertebrates and recognize PGN, the unique cell wall component of bacteria. In insects, PGRPs mainly activate antimicrobial pathways. Mammalian PGRPs are not involved in signaling pathways, but are bactericidal by interfering with PGN synthesis. A third group, PGN-hydrolyzing PGRPs, has been found in insects and mammals. Although soluble and membrane-bound variants of PGRPs with different molecular weights exist, all PGRPs possess a domain that closely resembles the AmiE fold. To date, structures of this domain from *Drosophila melanogaster* (PGRP-LB and PGRP-SA) and *Homo sapiens* (PGRP-IαC and PGRP-S) are known. All four PGRPs can be superimposed with AmiE with low r.m.s. deviations (Figure 8). Only PGRP-LB functions as a PGN-hydrolyzing amidase, whereas the other structurally known PGRPs can bind PGN but are unable to cleave it. Non-lytic PGRPs lack zinc-coordinating residues in the active site. They likely interfere with bacterial growth by enclosing parts of the PGN layer and thereby preventing further crosslinking [17].

The superposition of AmiE with PlyL and the PGRPs shows that the best agreement is seen around the putative PGN-binding groove and the active site (Figure 8B,C). The coordination of zinc in the amidases PGRP-LB and PlyL closely resembles the zinc-binding site in AmiE. The histidines surrounding the zinc ions are in equivalent positions, while D179 is replaced with cysteines in PGRP-LB and PlyL. Both aspartates and cysteines can ligate zinc. In addition, the carboxylate groups of E119 in AmiE and E90 in PlyL occupy similar positions. E90 has been suggested to act as a proton shuttle in the catalytic cycle of PlyL, similar to the proposed role of E119 in AmiE (Figure 3B). This role is taken over by the deprotonated side chain of Y78 in PGRP-LB, which protrudes from the adjacent α2-β3 loop, bringing its phenol oxygen in close proximity to the E119 carboxylate in the superposition (Figure 8B). The side chain of AmiE residue H177 lies near the side chain of K135 in PlyL. Both residues in the same position are predicted to stabilize a transition state during substrate cleavage.

All six structures also have a conserved asparagine at the end of their putative PGN-binding sites (Figure 8C). For the non-catalytic amidases, it has been shown [18] that an asparagine in this position contacts the carbonyl oxygens of D-iGln and L-Lys (PGRP-IαC, PGRP-SA) of MTP via hydrogen bonds. In the docking model of PGRP-S, only L-Lys is contacted by the corresponding asparagine. Our docking model of AmiE and MTP shows that N112 is engaged in hydrogen bonds with the D-iGln and L-Lys carbonyls (Figure 6). Both hydrogen bonds are likely critical for anchoring the substrate as we find that compounds that lack the third amino acid cannot be processed by AmiE.

As expected, regions more distant from the substrate-binding groove are structurally more diverse. AmiE and the N-terminal PlyL domain are modular proteins that are connected to additional domains that mediate interactions with the cell wall. AmiE mainly differs from PlyL by a unique 20 residue N-terminal extension, including helix α1. As this helix lies close to the C-terminus of AmiE (Figure 1A), it likely interacts with the repeats R1 and R2, probably forming an adapter between the catalytic domain and the cell wall binding domain. The PGRPs differ in number and arrangement of α-helices at their C-terminus from AmiE and PlyL. They also all have an additional 3_10_ helix, which is absent in the bacterial amidases. This helix is located in the so-called PGRP-specific segment, near the N-terminus, which serves to bind different non-PGN substrates such as effector molecules or molecules involved in signaling pathways.

## Discussion

We have solved the crystal structure of the AtlE amidase AmiE from *Staphylococcus epidermidis* at high resolution. This is not only the first structure of a staphylococcal amidase, but also of an amidase from any bacterium with a gram-positive cell wall architecture. Comparison with related bacterial enzymes reveals few conserved residues. Almost exclusively, these map to an elongated groove on one side of the protein. Architecture and size of the groove indicate that it is likely to serve as the binding site for a portion of PGN. A tetrahedrally coordinated zinc ion at the center of the groove marks the active site of the enzyme. Mutagenesis experiments at this site provide strong evidence that AmiE functions as a zinc-dependent metalloprotease.

Currently, fourteen protein structures that share the N-acetylmuramyl-L-alanine amidase-like fold observed in AmiE have been deposited in the Protein Data Bank. Five of these structures show catalytic activity, but so far none of the catalytically active enzymes has been crystallized in complex with a ligand. Therefore, the principles that guide the interaction of these enzymes with their substrates remain undefined. In order to provide a basis for understanding their ligand-binding and activity properties, we first defined MTP as the minimal ligand that can still be cleaved by AmiE, and then performed docking studies with this ligand. Our results indicate that the carbohydrate moiety plays a minor role in binding. However, the glutamine isoform in the second position and presence of a third amino acid in the peptide stem are key determinants of binding, needed to position the ligand in the binding groove. Furthermore, mutation of lysine in the third position to an alanine does not abolish substrate recognition by the enzyme, indicating that contacts with the third amino acid are restricted to the main-chain of the peptide stem. This finding is consistent with our docking results. The digestion assays, using substrate derivatives with one, two and three glycines residues attached to the lysine side chain, show that all three substrates are processed equally well, but show a significantly higher binding affinity than the alanine derivative, which is likely due to additional van der Waals interactions with either the lysine side chain or the first glycine residue.

What are the key requirements for successful PGN binding and cleavage by AmiE? Replacing MurNAc with a fluorophore, changing the amino acid in the third position, and absence of the D-iGln amidation [12] clearly do not prevent binding, and thus variations and extensions at these positions can likely be tolerated by the enzyme. However, the presence of the glutamine isoform at position two of the peptide is likely critical for binding. It was previously shown that a three amino-acid substrate with a glutamine in its standard configuration is not recognized by AmiE [12]. Our docking experiments clearly show that the D-iGln residue forms the majority of hydrogen bonds with AmiE, supporting a critical role for this residue. Moreover, a standard glutamic acid at the equivalent position would not be accommodated by AmiE, as its side chain would clash with residues lining the groove. The results from our docking studies are also consistent with structural information on a complex of PGRP-IαC with PGN and docking models of PGRP-S and PGRP-SA. All of these show extensive contacts with D-iGln [18]. The digestion assay with an alanine substrate clearly showed that the absence of the lysine side chain has only a minor impact on substrate recognition when compared to the Lys(Gly)_x_ substrates. Our docking model, in which the main chain of the third amino acid is primarily involved in interactions, supports these data. The main chain of the third amino acid can therefore be described as a minimal motif that is essential for recognition of the substrate.

N112, which is located towards the lower end of the groove, is strictly conserved in bacterial amidases (Figure 2A). We were surprised to find an asparagine at an equivalent position in catalytically active as well as inactive PGRPs (Figure 8C). This residue is the only conserved amino acid among bacterial and eukaryotic amidases that participates in PGN binding. Its conservation therefore indicates that it is required for the recognition of a muramylpeptide with a three-peptide stem. In our docking model, the δ2 nitrogen of N112 forms hydrogen bonds with the main chain carbonyl oxygens of D-iGln and L-Lys. We therefore postulate that a common PGN binding mode is shared by all proteins with an N-acetylmuramyl-L-alanine amidase-like fold.

Our data show that only a small number of PGN consensus motifs are required for recognition by AmiE. Such motifs are present in many different types of cell walls. How does the amidase then ensure species-specific binding to the bacterial cell wall, and how does this compare with other amidases that have similar catalytic activities but act on different cell wall types? It is likely that AmiE uses a dual strategy to ensure species-specific substrate recognition with high affinity. In the first step, the repeating units that follow AmiE in sequence (Figure 1B) likely mediate cell wall binding and help to position the catalytic domain correctly. Such a cell wall anchoring function has been described for the *S. epidermidis* AtlE repeats R1R2 [7] as well as for the repeat domains of PlyL from a *B. anthracis* prophage [16]. The repeat domains are highly positively charged and might interact with the zwitterionic backbone of lipoteichoic acids on the cell surface [19]. Upon initial attachment via the repeat domains, the catalytic domain would then be close enough to the PGN substrate to bind and cleave it at the appropriate location.

All bacterial cell walls share common PGN motifs recognized by amidases, while other motifs are more variable among different species. Among those are the amidation of the D-iGln side chain, the variable third amino acid in the stem, and the number and type of amino acids in the interpeptide bridge. All these variations likely increase amidase affinity to specific cell walls. It has previously been shown that lack of the D-iGln amidation results in a lower binding affinity to AmiE [12]. Nothing is known about either the mode of interaction between repeat domains and lipoteichoic acids or the influence of the interpeptide bridge on binding of the PGN-substrate. This bridge, which consists of five glycines in *S. aureus* and various other staphylococcal species, links the third and fourth amino acids of two neighboring PGN-strands and is responsible for crosslinking. Number and type of amino acid are highly variable among different species and could therefore play a crucial role for species-specific recognition. Further investigations will be necessary to complete the picture concerning the interactions between AmiE, the repeat domains and their substrates.

It has been clearly demonstrated that proper function of autolysins is essential to allow for normal cell division and therefore growth of Staphylococci. We show here that alterations in the active center of AmiE directly affect cell proliferation and therefore are likely to prevent spreading of a Staphylococci infection. The structure-function analysis presented here therefore provides the framework for understanding a key step in the function of all staphylococcal autolysins: the recognition, selectivity, and catalytic mechanism of cleavage of a PGN fragment. This information should therefore guide efforts to design specific inhibitors that can block autolysin function and thus prevent staphylococcal growth.

## Materials and Methods

### Protein expression and purification

A DNA fragment coding for amino acids 303 to 516 of the *atlE* gene product was amplified via PCR from *S. epidermidis* O-47 [20] genomic DNA and cloned into the pGEX 4T-3 expression plasmid (GE Healthcare) using *Bam*HI and *Xho*I restriction sites. The protein was expressed in *Escherichia coli* BL21 (DE3) (Stratagene) fused to a thrombin cleavable N-terminal GST-tag. Bacteria were grown at 37°C in LB-medium supplemented with 50 µg mL^-1^ ampicillin until the OD_600_ had reached ∼0.4. Expression of the fusion protein was induced with 1 mM isopropyl-β-D-1-thiogalactopyranoside (IPTG). The temperature was then reduced to 25°C, and growth of the culture continued for 6 h. Cells were harvested and resuspended in lysis buffer (50 mM Tris/HCl pH 8.0, 150 mM NaCl, 1 mM Phenylmethylsulfonylfluoride (PMSF). After lysis, the mixture was centrifuged for 30 min at 50000xg to remove insoluble material. The soluble fraction was passed through a 0.45 µm filter and applied to a 5 ml GSTrap FF column (GE Healthcare). After washing, the protein was released from the GST-tag by on-column cleavage with 10 units thrombin/mg fusion protein. The eluate was concentrated and applied to a Superdex 75 16/60 gel filtration column (GE Healthcare), again using the lysis buffer (without PMSF). The purified enzyme was >95% pure as judged by SDS-PAGE, and was used for crystallization.

### Crystallization and structure determination

Purified AmiE (20 mg/ml) was mixed in a 1∶1 ratio with crystallization buffer containing 22% (v/v) 1,4-butanediol, 0.1 M imidazole pH 7.0 and 0.15 M zinc acetate. Crystals grew at 4°C using the hanging drop method. Prior to freezing in liquid nitrogen, the crystals were placed in crystallization solution supplemented with 20% glycerol as cryoprotectant. For phase determination, crystals were soaked for 5 min in crystallization solution supplemented with 10 mM samarium chloride before cryoprotection and freezing. Crystals of AmiE belong to space group P4_3_2_1_2 with two molecules in the asymmetric unit (Table 1). Native data were collected at beamline X06S at the Swiss Light Source (Villigen, Switzerland) using a Mar225 CCD detector. Data collection of derivatized crystals was performed using a Rigaku Micromax 007 HF rotating anode X-ray generator and a Mar345 dtb detector. Data were indexed, integrated and scaled using the XDS package [21] and the HKL software package [22]. Phases were determined by single isomorphous replacement. Native and derivative data were scaled with Scaleit [23]. Heavy atom sites were identified by manual inspection of difference Patterson maps calculated with fft [23] and refined using MLPhaRe [23]. The initially obtained phases were improved by density modification as implemented in DM [23]. At this point, secondary structure elements could clearly be identified in the electron density map. Non-crystallographic averaging (DM & O) [23],[24] further improved the density. Model building was performed manually using Coot [25]. The structure was refined to 1.7 Å resolution with CNS [26],[27] and Refmac5 [23]. The final model comprises amino acids 7–214. Although present in the purified protein, the six N-terminal residues are not visible in the electron density map and were therefore not built. The structure also contains 14 zinc ions per monomer. Coordinates and structure factors have been deposited with the Protein Data Bank with the accession code 3LAT. A second crystal form, which could be obtained from a zinc free crystallization condition, contains only the zinc ion in the active site. Data for this crystal form were collected at the ESRF (Grenoble, France).

**Table 1:** Data collection and structure refinements statistics.

	Native	SmCl_3_
**Data collection**		
Space group	P4_3_2_1_2	P4_3_2_1_2
Cell dimensions (Å)	99.45, 99.45, 148.78	99.57, 99.57, 149.15
Wavelength (Å)	0.9999	1.5418
Resolution (Å)	40.0–1.70 (1.76–1.70)	40.0–2.30 (2.38–2.30)
R_sym_ *	0.09 (0.33)	0.08 (0.32)
I/σI	19.6 (2.40)	18.8 (1.92)
Completeness (%)	99.4 (99.4)	80.2 (78.2)
Redundancy	10.0 (6.2)	1.8 (1.9)
Total reflections	824913	50047
Unique reflections	82089	27285
**Refinement**		
Resolution (Å)	30.8–1.7	
R_work_/R_free_ #	0.143/0.167	
Number of atoms		
Protein	3408	
Ligand/ion	28/15	
Water	523	
B-factors (Å^2^)		
Protein	25.8	
Ligand/ion	33.4/25.7	
Water	46.7	
r.m.s. deviations		
Bond lengths (Å)	0.009	
Bond angles (°)	1.150	
Ramachandran plot (%)		
Most favored region	96.01	
Additionally allowed region	3.99	

r.m.s, root-mean-square. Values in parentheses correspond to highest resolution bin.

*R_sym_ = Σ| |F_obs_(hkl) | - |F_calc_ (hkl) | |/Σ |F_obs_ (hkl)

# R-factor = Σ | *I* - <*I*> |/Σ*I*

### Construction of amidase mutants, and complementation assay

Amino acids H60, H177 and D179 were each mutated to alanine by site directed mutagenesis using the QuikChange II XL Kit (Stratagene) with primer pairs H60A_5′ (5′-GAAGGTATCGTTGTTGCTGATACTGCAAATGATA-3′), H60A_3′ (5′-TATCATTTGCAGTATCAGCAACAACGATACCTTC-3′), H177A_5′ (5′-GGAGGT ACTGATGCTGCTGACCCTCACC-3′), H177A_3′ (5′-GGTGAGGGTCAGCAGCAT CAGTACCTCC-3′), D179A_5′ (5′-CTGATCACGCTGCTCCTCACCAA TATTTAAG-3′), and D179A_3′ (5′-CTTAAATATTGGTGAGGAGCAGCGTGATCAG-3′). The pGEX 4T-3 expression plasmid carrying the *amiE* gene fragment was used as the template. The mutated plasmids were then used for protein overexpression in *E. coli*. In addition, the mutated amidase genes were assayed with respect to their ability to complement the *S. aureus* SA113Δ*atlA* mutant *in vivo*. Therefore, the same mutations were introduced into the *E. coli/Staphylococcus sp*. shuttle vector pRC20 [4]. Briefly, pRC20 containing the mutated *amiE*-R_1,2_ gene was isolated from *E. coli* DH5α and transformed first into *S. aureus* RN4220 and subsequently into *S. aureus* SA113Δ*atlA* by electroporation. Transformants in *S. aureus* were selected with chloramphenicol. The DNA sequence was verified using amidase-specific primers. For the complementation assay, mutant cells were allowed to grow for 10 h in 50 ml liquid medium at 37°C under shaking. Aliquots were transferred into plastic tubes, allowed to settle for 2 min and photographed against a dark background.

### ELISA assay

ELISA assays were carried out with MTP-Biot. Greiner Microcolon plates were incubated for 24 h with 100 µl protein solution at a concentration of 10 µg/ml in PBS (pH 7.3) at 4°C. All following steps were done at 20°C. All washing steps were repeated three times with 250 µl wash buffer (0,05% (v/v) Tween 20 in PBS). Surfaces were blocked for 1 h with 250 µl milk powder (4% (w/v) in PBS), followed by a wash step. The coated plates were then incubated for 30 min with 100 µl of MTP-Biot solution (12,5 µg/ml in PBS). After washing, the wells were incubated for 1 h with Streptavidin linked to horseradish peroxidase solution. Unbound Streptavidin was removed by washing. For detection, 100 µl of 3,3′, 5,5′-tetramethylbenzidine solution was added to the wells for 3–5 min until a slight blue color appeared. The reaction was stopped by adding 100 µl 1 M H_2_SO_4_. Products were detected at 450 nm using an ELISA-reader (Thermo Multiscan).

### Synthesis of MTP compounds

Compounds MTP and MTP-Biot were synthesized as previously described [13] using standard solid phase synthesis protocols. Protection groups that rely on strong acids for cleavage were avoided. All peptides were synthesized by a solid-phase technique, using the fluoren-9-ylmethoxycarbonyl (Fmoc) strategy on a Syro-II-synthesizer (MultiSynTech). Fmoc-amino acids were purchased from Novabiochem, from MultiSynTech or from Iris Biotech. 2-(7-Aza-1H-benzotriazole-1-yl)-1,1,3,3-tetramethyluronium hexafluorophosphate (Applied Biosciences) was used as a coupling agent. The sugar moiety was obtained as Benzyl N-acetyl-4,6-O-benzylidenemuramic acid (Sigma). The benzyl group was removed by incubation with ammonium formiate at 40°C for 24 h in the presence of 10% Pd on charcoal (Acros organics). Purity and molecular mass of the product was analyzed by liquid chromatography mass spectrometry (LCMS) using a reversed phase C8 column for separation and ESI-MS for mass determination.

### MTP digestion assays

For each assay, 10 µg of wt or mutant protein and 0.1 mg of the appropriate ligand were incubated in 10 µl gel filtration buffer (50 mM Tris/HCl pH 8.0, 150 mM NaCl). The reaction was stopped after 72 h by adding 100 µl acetonitrile with 0,05% (v/v) formic acid. The sample was then frozen at -20°C until used for ESI-MS.

### Docking

The Schrodinger Suite [28],[29],[30] was used to perform docking simulations. In order to obtain a starting point for the docking, the only two available PGRP structures with a bound ligand (PDB codes: 2aph, 1twq) were superimposed onto the AmiE wt structure. The structure 1twq contains MTP as a ligand, while 2aph contains an MTP-based compound that is extended by two D-alanine residues at the C-terminus. Superposition was in each case performed using a least-squares fit algorithm in Chimera [31]. The superimposed structures were used to define a starting position and the boundaries of the docking grid. Since our own studies had shown that MTP is the minimal ligand for AmiE, we generated 64 MTP conformers using the docking algorithm implemented in the program Glide [28],[29] in the Schrodinger Suite. The docking was performed in two steps. A constrained initial docking using the XP algorithm was performed first, followed by an unconstrained QM-polarized docking protocol [32]. During the initial docking, the carbonyl atom in the peptide bond linking N-acetylmuramic acid to alanine was constrained to stay near the zinc ion in the active site. All solutions from this initial docking were then redocked using the unconstrained QM-Polarized Ligand Docking protocol. All results from the second docking protocol were similar with regard to their energy values. Therefore, each solution was inspected visually. Eight out of the ten reported solutions showed twisted conformations or unreasonable orientations in the binding cleft, and they were therefore not considered further. The remaining two solutions differ primarily in the orientation of the carbohydrate. The solution presented here has more hydrogen bonds and was therefore considered to be more likely.

### Synthesis of substrate derivatives

All peptides (Mca-Ala-D-iGln-Lys-D-Ala-Arg-OH, Mca-Ala-D-iGln-Lys(Gly)-D-Ala-Arg-OH, Mca-Ala-D-iGln-Lys-(Gly)2-D-Ala-Arg-OH, Mca-Ala-D-iGln-Lys(Gly)3-D-Ala-Arg-OH, Mca-Ala-D-iGln-Ala-D-Ala-Arg-OH) were synthesized by a solid-phase technique, using the fluoren-9-ylmethoxycarbonyl (Fmoc) strategy on a Syro II synthesizer (MultiSynTech, Witten, Germany). Peptide synthesis was performed according to Lützner et al. [12]. In contrast to the original protocol, the fluorescence of the N-terminal Mca-group is not quenched. Since the side chain of the alanine substrate is not long enough to bring an attached quencher-group in close proximity to the fluorophore it was left also left out for the other peptides. The C-terminal arginine was attached for reasons of solubility.

For the incorporation of the glycine at the side chain of lysine, a Fmoc-Lys(Dde)-OH protected derivative was used. After the final deprotection of the Fmoc group, the free amino group was reacted with di-*tert-*butyl-dicarbonate. The Dde group was cleaved with 3% of hydrazine and then manually coupled with glycine. Fmoc-amino acids were purchased from Novabiochem (San Diego, CA, USA), from MultiSynTech or from Iris Biotech (Marktredwitz, Germany). Mca was obtained from Sigma (Deisenhofen, Germany). 2-(1H-benzotriazole-1-yl)-1,1,3,3-tetramethyluronium–tetrafluoroborate (TBTU) and 1-hydroxybenzotriazole (HOBt) were from MultiSynTech. The final deprotected peptides were purified by semi-preparative reversed phase (RP) HPLC, using a Reprosil C-8 column (5 µm particle size, 150×10 mm) from Dr. Maisch GmbH (Tuebingen, Germany) and a two solvent system: (A) 0.055% (v/v) trifluoroacetic acid (TFA) in water and (B) 0.05% (v/v) TFA in 80% (v/v) ACN in water. Purity and molecular mass of the collected fractions was analyzed by matrix-assisted laser-desorption ionization–MS (MALDI–MS), using a MALDI time-of-flight system (Reflex IV, Bruker Daltonics, Bremen, Germany). Substrates were dissolved in DMSO and stored as 2 mM stock solutions at -20°C.

### Digestion assays with substrates derivatives

The five substrates, each at a concentration of 70 µM, were processed by 7.2 µM AmiE at 37°C in digestion buffer (50 mM sodium phosphate buffer, pH 7.2). After 1, 3 and 6 h, the reaction was stopped by adding trifluoroacetic acid to a final concentration of 1%. Decrease of the initial substrate and formation of the Mca product were analyzed by analytical RP-HPLC using a binary HPLC system (Shimadzu, Tokyo, Japan) with a UV detector, a Nucleosil C-18 column (5 µm particle size, 150×2 mm) from Dr Maisch GmbH (Tuebingen, Germany) and a two solvent system: (A) 0.055% (v/v) TFA in water and (B) 0.05% TFA in 80% acetonitrile in water. The substrate was well separated from the generated cleavage product Mca, and was quantified at 214 nm.

### Zymogram analysis, lysis assay and peptidoglycan purification

Bacteriolytic enzyme profiles were obtained with zymograms and lysis assays. The cell wall lytic activity of recombinant proteins purified from *E. coli* was analyzed in 12% (v/v) polyacrylamide gels with heat-killed cells of *S. aureus* SA113 embedded at a concentration of 0.2% (w/v) [4]. Protein concentration was measured using the Sigma protein detection kit with bovine serum albumin as standard. To gain a higher contrast, the gels were stained with 0.1% (w/v) methylene blue for 5 min and washed until clear bands became visible. Peptidoglycan was isolated from stationary phase cultures of *S. aureus* SA113 [33]. Briefly, cells were harvested by centrifugation and boiled for 60 min in 4% (w/v) SDS. After washing with H_2_O_dest_, the cell wall fragments were incubated with 0.5 mg ml^−1^ trypsin for 16 h at 37°C to degrade cell-bound proteins. After centrifugation and washing with water, the cell walls were incubated for 5 h with 10% TCA to remove teichoic acids. For a quantitative analysis of lysis, purified peptidoglycan of *S. aureus* SA113 was dissolved in 1 ml 100 mM sodium phosphate buffer and adjusted to OD_578_ = 0.3. The insoluble PGN was mixed with 20 µg of purified enzyme. Cell lysis was measured as the decrease in OD_578_ in a spectrophotometer.
